# Orthodontic treatment in a patient with unilateral open-bite and Becker muscular dystrophy. A 5-year follow-up

**DOI:** 10.1590/2176-9451.19.6.037-045.oar

**Published:** 2014

**Authors:** Juan Fernando Aristizabal, Rosana Martínez Smit

**Affiliations:** 1 Universidad del Valle, Colombia, Assistant Professor and Head of the Department of Orthodontics, Universidad del Valle, Colombia; 2 CES University, Department of Orthodontics, Colombia, Assistant Professor, Department of Orthodontics, CES University, Colombia

**Keywords:** Muscular dystrophies, Corrective Orthodontics, Open bite

## Abstract

**INTRODUCTION:**

Becker muscular dystrophy is an X-chromosomal linked anomaly characterized by progressive muscle wear and weakness. This case report shows the orthodontic treatment of a Becker muscular dystrophy patient with unilateral open bite.

**METHODS:**

To correct patient's malocclusion, general anesthesia and orthognathic surgery were not considered as an option. Conventional orthodontic treatment with intermaxillary elastics and muscular functional therapy were employed instead.

**RESULTS:**

After 36 months, open bite was corrected. The case remains stable after a 5-year post-treatment retention period.

## Abstract (pt)

**INTRODUÇÃO:**

a distrofia muscular de Becker é uma anomalia ligada ao cromossomo X, caracterizada por desgaste muscular progressivo e fraqueza. Este relato de caso mostra o tratamento ortodôntico de um paciente com distrofia muscular de Becker e mordida aberta unilateral.

**MÉTODOS:**

na correção de sua má oclusão, anestesia geral e cirurgia ortognática não foram consideradas como uma opção. Tratamento ortodôntico convencional com elásticos intermaxilares e terapia miofuncional foram empregados.

**RESULTADOS:**

após 36 meses, a mordida aberta foi corregida. O processo manteve-se estável após um período de cinco anos de retenção de pós-tratamento.

## INTRODUCTION

Muscular dystrophies are part of a variety of genetic alterations associated with diverse gene mutations that lead to muscular weakness and dystrophy.^1^

Similarly to Duchenne progressive muscular dystrophy, Becker muscular dystrophy is an X-chromosomal linked anomaly characterized by progressive muscle wear and weakness only present in men.^2^^,^ 3

It has been reported that Duchenne progressive muscular dystrophy affects about 4,000 newborn males, whereas Becker muscular dystrophy only affects about 10% of these cases. Both types of patients have difficulty walking and delayed motor skills during the early stages of life. Also, they present myopathy that worsens progressively with age, which in the future will affect their breathing and circulation.^4^

Becker dystrophy differs from Duchenne dystrophy, since the former presents a partially functional peptide called dystrophin. This is why Becker dystrophy is of slower progression and muscles are mildly affected. Additionally, life expectancy is longer than in patients with Duchenne dystrophy, in which case dystrophin is completely absent.^5^^,^ 6

The literature has proved that patients with Duchene dystrophy usually have severe anterior open bite with an inclined mandibular plane.^7^^,^ 8 ^,^ 9 However, there are no reports describing the occlusal and skeletal characteristics of patients with Becker dystrophy. There is also lack of orthodontic treatment reports on these two kinds of dystrophies, probably because these patients suffer of severe occlusal and masticatory problems.

Nowadays, there is only one case reported in the literature in which a patient with Becker dystrophy is orthodontically treated. This patient has similar characteristics to subjects with Duchenne dystrophy, namely: inclined mandibular plane, counterclockwise rotation of the mandible and increased gonial angle.^10^

This article reports the case of a patient with Becker muscular dystrophy and unilateral open bite subjected to orthodontic therapy.

## DIAGNOSIS AND ETIOLOGY

The patient was a male born to healthy parents. He had difficulty walking since his early childhood. Muscular biopsy and a genetic blood test were carried out and he was diagnosed with Becker muscular dystrophy.

**Figure 1.**
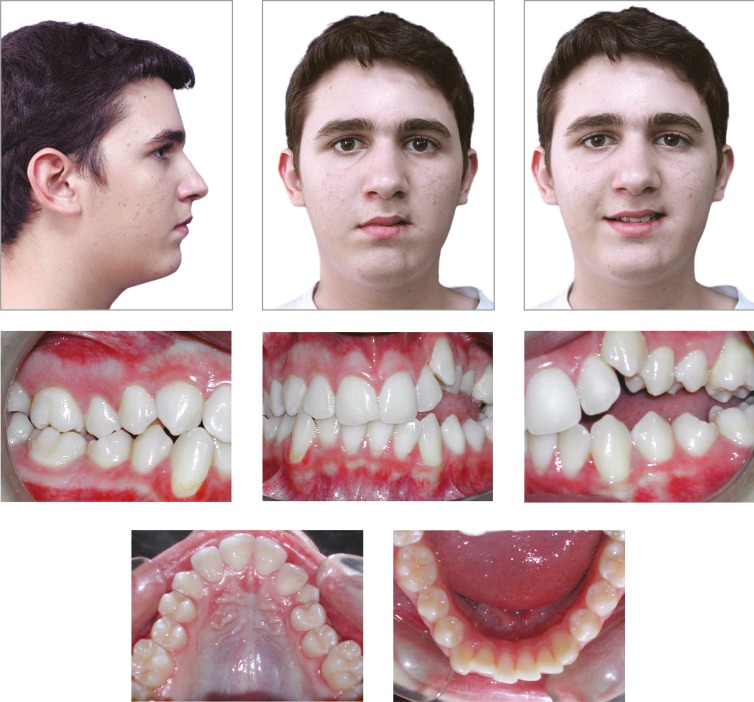
Facial and intraoral pretreatment photographs.

**Figure 2.**
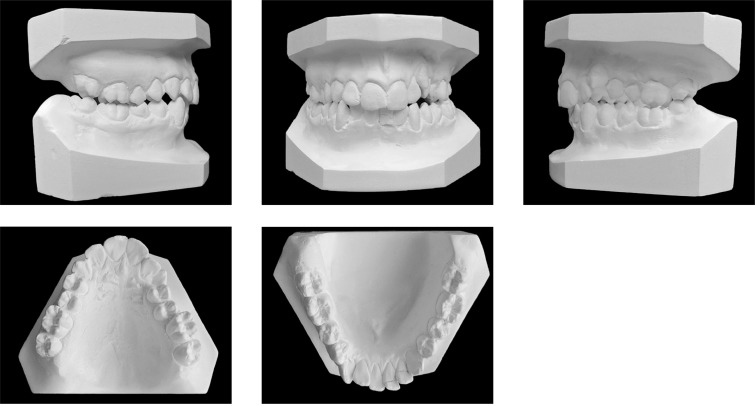
Pretreatment dental casts.

**Figure 3.**
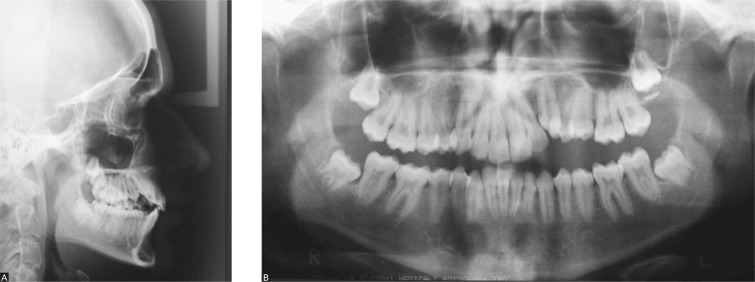
Pretreatment radiographs. A) Cephalometric radiograph; B) panoramic radiograph

The patient was 14 years and 3 months old at the initial orthodontic appointment. He had a straight profile and muscular hypotonia (Fig 1). He had difficulty walking and delayed motor skills. His chief complaints were crowding and difficulty chewing food (Figs 1 and 2). His skeletal pattern was Class I with mild maxillary retrusion, neutral mandibular rotation, maxillary and mandibular incisors with good angulations (Fig 2 and Tab 1), and retained and poorly positioned third molars (Fig 3B). Clinical examination revealed open bite on the left side combined with abnormal tongue posture, cross bite between #13 and 43 and a collapsed upper arch with crowding.

**Table 1.** Cephalometric measurements.

Measurement	Mean	Pretreatment values	Post-treatment values	5-year follow-up
SNA (degrees)	76.2 - 83.8	76	77	80
SNB (degrees)	75 - 81	74	76	77
ANB (degrees)	-0.5 ± 5.1	2	1	3
FH-MP (degrees)	17 - 28	21	20	20
LAFH (mm)	66.7 - 74.6	71	73	73
U1-FH (degrees)	105 - 115	113	119	115
L1-MP (degrees)	81.5 - 97	96	100	110

## TREATMENT OBJECTIVES

The following treatment objectives were established: (1) Correct unilateral open bite; (2) Improve overjet and overbite; (3) Correct crowding; (4) Improve patient's masticatory function and facial esthetics.

## TREATMENT ALTERNATIVES

There were some different treatment alternatives to correct patient's malocclusion. The first treatment option to correct unilateral open bite was surgical; however, due to potential complications during general anesthesia, this alternative was dismissed. Another option was treating the patient with orthodontic fixed appliances and myofunctional therapy in order to improve muscular hypotonia. This last option was chosen for the patient reported herein.

## TREATMENT PROGRESS

**Figure 4.**
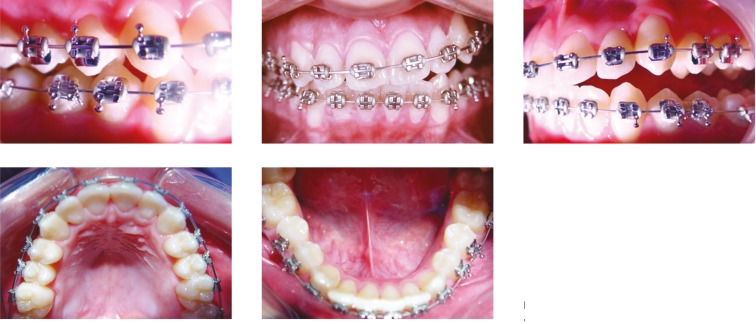
Intraoral photographs at the end of alignment and leveling phase.

**Figure 5.**
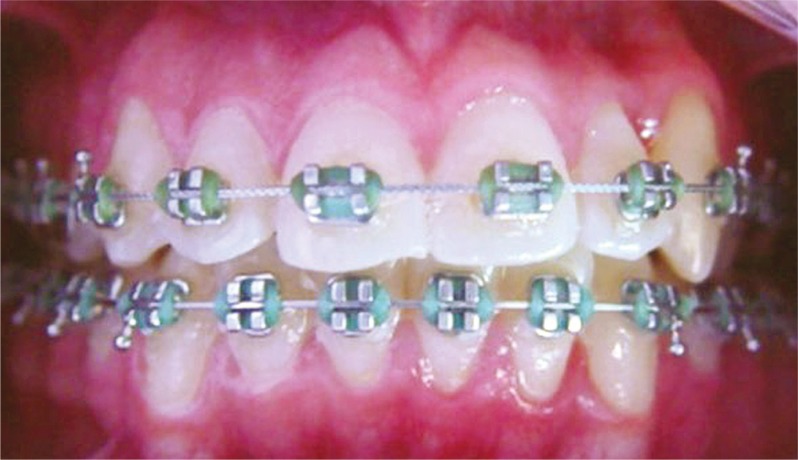
Intraoral photograph at the end of the use of vertical elastics.

Clinicians decided to start with non-extraction orthodontic treatment using Orthos^TM^ brackets (Ormco Corp. Orange, CA, USA) bonded from second molar to second molar. Brackets on the upper left side had a more gingival position so as to promote extrusion. After aligning and leveling (Fig 4), the patient started to use vertical elastics (1/4-in, 3.5 oz.) and a maxillary appliance to avoid tongue interference. Great improvement in the correction of open bite was observed. The use of elastics continued until good posterior occlusal contact was achieved (Fig 5). Mechanics included a normal archwire sequence, starting with cooper NiTi 0.014-in, followed by cooper 0.016 x 0.022-in and finishing with turbo wire 0.017 x 0.025-in. After open bite correction, right Class II and left Class III elastics were used for two weeks. Elastics were suspended for six weeks to assess stability.

**Figure 6.**
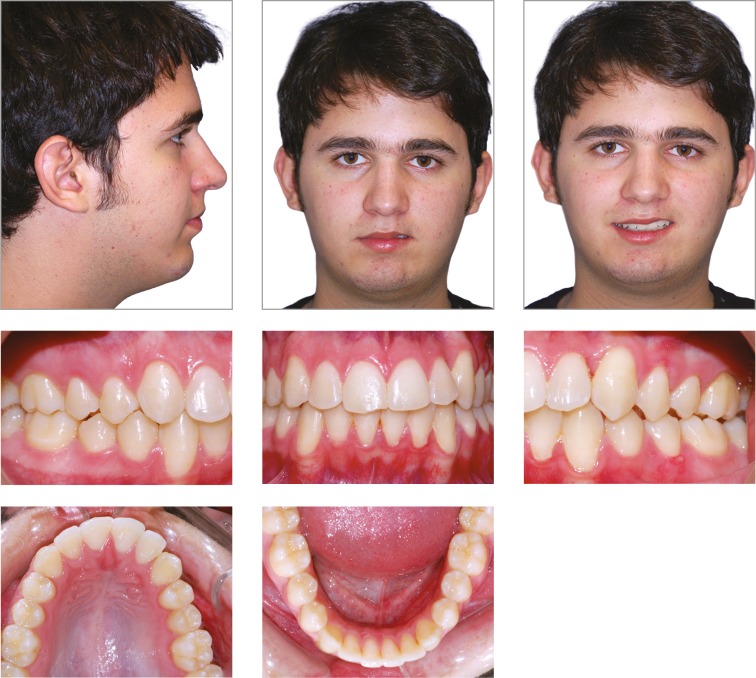
Facial and intraoral post-treatment photographs.

**Figure 7.**
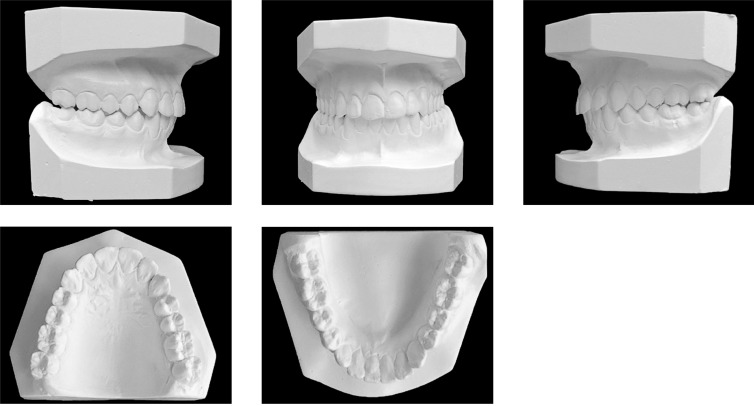
Post-treatment dental casts.

**Figure 8.**
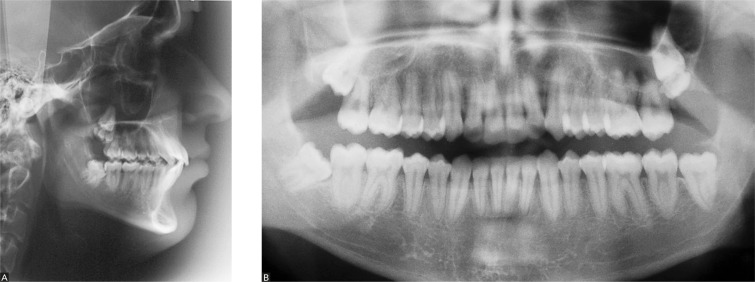
Post-treatment radiographs. A) Cephalometric radiograph; B) Panoramic radiograph.

**Figure 9.**
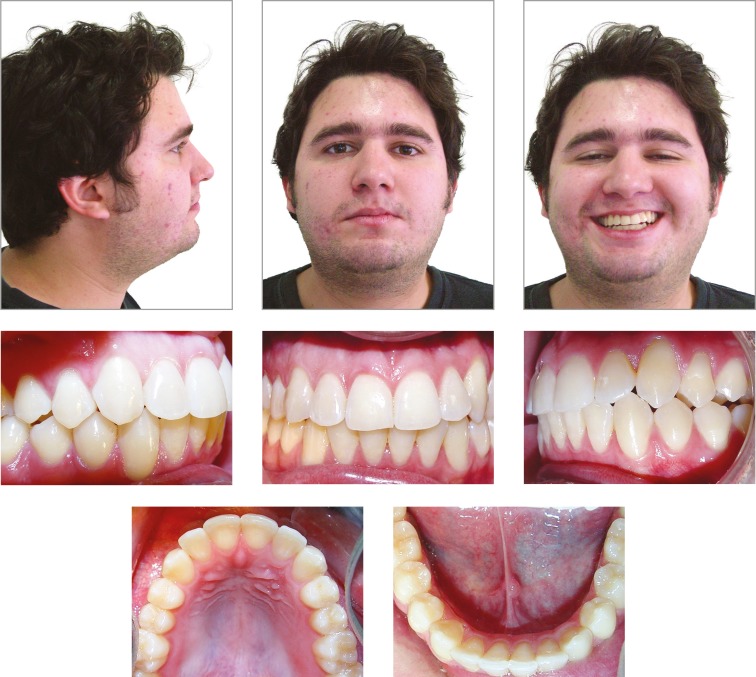
Facial and intraoral photographs after 5 years in retention.

**Figure 10.**
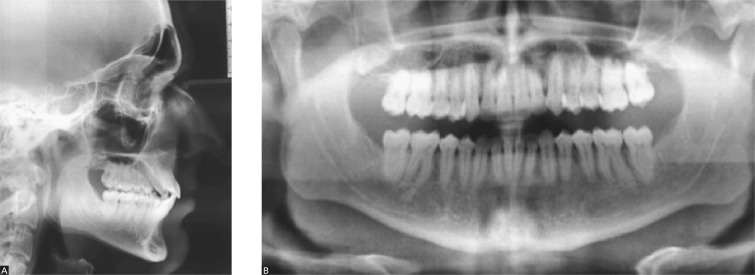
Radiographs after 5 years in retention. A) Cephalometric radiograph; B) Panoramic radiograph.

Vertical stability was achieved. For this reason, after 35 months of treatment, it was decided to remove the maxillary appliances and install a Hawley retainer. In the following month, mandibular brackets were removed and the same retention protocol was implemented (Figs 6, 7 and 8). The patient was referred to myofunctional therapy and extraction of third molars. He was reevaluated after five years in retention and a slight relapse of unilateral open bite was observed (Fig 9). Inclination of maxillary and mandibular incisors was different between post-treatment and retention phases (Fig 10 and Tab 1).

## DISCUSSION

Previous case reports presented Duchenne muscular dystrophy associated with severe open bite, wide arches, inclined mandibular plane and increased gonial angle.^7^^,^ 8 ^,^ 9 The patient reported herein had significant malocclusion despite the fact that Becker muscular dystrophy affects muscles in a slower and milder manner than Duchenne muscular dystrophy.^5^^,^ 6

Perhaps, this malocclusion needed a treatment plan that included a surgical approach, but there is evidence supporting that dealing with these types of patients under general anesthesia can be complex, particularly due to potential complications such as heart failure,^11^^,^ 12 ^,^ 13 malignant hyperthermia and rhabdomyolysis.^15^^,^ 16

As reported herein, the patient with Becker muscular dystrophy had orthodontic treatment completed within 36 months. There are possible factors that contributed to extend treatment time, namely: hypotonia of closure muscles, tongue interference and patient's compliance.

This case showed stability after five years in retention (Fig 8) mainly due to myofunctional therapy. Reports have proven that this kind of therapy increase masticatory muscle activity and produce forward mandibular rotation in patients with open bite.^17^ Furthermore, this patient had good compliance during the retention phase.

As observed in this case report, open bite caused by Becker muscular dystrophy can be corrected with fixed appliances complemented by myofunctional therapy, without the need for surgery. Positional changes are expected over time due to the complex muscular dynamics of these patients that are always against normal perioral balancing forces, as shown by this specific case with inclination of both incisors (Fig 10 and Tab 1).

## CONCLUSIONS

" Orthodontic treatment and myofunctional therapy are important tools to restore occlusal and functional balance in patients with Becker muscular dystrophy and associated malocclusion.

" Good control during the retention phase is vital for treatment outcomes stability in these patients.
